# Concepts of mental capacity for patients requesting assisted suicide: a qualitative analysis of expert evidence presented to the Commission on Assisted Dying

**DOI:** 10.1186/1472-6939-15-32

**Published:** 2014-04-22

**Authors:** Annabel Price, Ruaidhri McCormack, Theresa Wiseman, Matthew Hotopf

**Affiliations:** 1 Clinical Research Worker in Palliative Care Psychiatry, King’s College London, Institute of Psychiatry, 10 Cutcombe Road, London SE5 9RJ, UK; 2 ST3 Academic Clinical Fellow Psychiatry, King’s College London, Institute of Psychiatry London, UK; 3 Strategic Lead for Health Service, The Royal Marsden NHS Foundation Trust, Fulham Road, London SW3 6JJ, UK; 4 Professor of General Hospital Psychiatry, King’s College London, Institute of Psychiatry London, UK

**Keywords:** Assisted suicide, Mental capacity, Qualitative

## Abstract

**Background:**

In May 2013 a new Assisted Dying Bill was tabled in the House of Lords and is currently scheduled for a second reading in May 2014. The Bill was informed by the report of the Commission on Assisted Dying which itself was informed by evidence presented by invited experts.

This study aims to explore how the experts presenting evidence to the Commission on Assisted Dying conceptualised mental capacity for patients requesting assisted suicide and examine these concepts particularly in relation to the principles of the Mental Capacity Act 2005.

**Methods:**

This study was a secondary qualitative analysis of 36 transcripts of oral evidence and 12 pieces of written evidence submitted by invited experts to the Commission on Assisted Dying using a framework approach.

**Results:**

There was agreement on the importance of mental capacity as a central safeguard in proposed assisted dying legislation. Concepts of mental capacity, however, were inconsistent. There was a tendency towards a conceptual and clinical shift toward a presumption of incapacity. This appeared to be based on the belief that assisted suicide should only be open to those with a high degree of mental capacity to make the decision.

The ‘boundaries’ around the definition of mental capacity appeared to be on a continuum between a circumscribed legal ‘cognitive’ definition of capacity (in which most applicants would be found to have capacity unless significantly cognitively impaired) and a more inclusive definition which would take into account wider concepts such as autonomy, rationality, voluntariness and decision specific factors such as motivation for decision making.

**Conclusion:**

Ideas presented to the Commission on Assisted Dying about mental capacity as it relates to assisted suicide were inconsistent and in a number of cases at variance with the principles of the Mental Capacity Act 2005. Further work needs to be done to establish a consensus as to what constitutes capacity for this decision and whether current legal frameworks are able to support clinicians in determining capacity for this group.

## Background

Assisting suicide remains illegal in England and Wales following three unsuccessful attempts to pass Bills to legalise the practice in 2003 [1], 2004 [2] and 2005 [3]. In 2012 a new draft Bill *‘Safeguarding Choice: A Draft Assisted Dying Bill for Consultation’*[4] was published, and in May 2013 a new Assisted Dying Bill to ‘*Enable competent adults who are terminally ill to be provided at their request with specified assistance to end their own life; and for connected purposes*’ was tabled in the House of Lords by Lord Falconer [5]. The Bill is currently scheduled for a second reading in the House of Lords in May 2014. Mental capacity is once again proposed as a key safeguard and the Bill stipulates that the Secretary of State may issue one or more codes of practice in connection with *‘assessing whether someone has the capacity to make such a decision’* and ‘*recognising and taking account of the effects of depression or other psychological disorders that may impair a person’s decision-making’*. Mental capacity is construed in the Bill in accordance with the Mental Capacity Act 2005 [6].

Prior to the drafting of the new Bill, the “Commission on Assisted Dying”, hosted by DEMOS (a think tank focussed on power and politics) [7], funded by author Sir Terry Pratchett and businessman Bernard Lewis (both proponents of assisted dying) and chaired by Lord Falconer was convened in September 2010. Its stated aims were to consider whether the current legal and policy approach to assisted dying in England and Wales was *‘fit for purpose’* and to *‘explore the question of what a framework for assisted dying might look like, if such a system were to be implemented in the UK, and what approach to assisted dying might be most acceptable to health and social care professionals and to the general public’*[8]. After gathering evidence, the Commission published its findings in early 2012 entitled *‘The current legal status of assisted suicide is inadequate and incoherent’* and recommended the provision of the choice of assisted dying for mentally competent adults with terminal illness [9]. The Commission proposed eligibility criteria to be met in order to proceed with a request for assisted dying. These comprised (i) the presence of terminal illness; (ii) that the decision should be voluntary; and (iii) that *“(t)he person has the mental capacity to make a voluntary and informed choice, and the person’s decision making is not significantly impaired as a result of mental health problems such as depression”.*

In line with the new Bill, the report emphasised establishment of mental competence as a central safeguard in any legal process allowing assisted suicide. The Commission concluded that assessment of mental capacity for every eligible patient requesting assisted suicide should be undertaken, primarily by doctors, and that the relevant professional bodies should be responsible for developing a code of practice for the assessment of mental capacity. The Mental Capacity Act 2005 was invoked as the framework within which mental capacity should be assessed. The Act, which sets out criteria for a test of capacity aims to help clinicians to preserve patient autonomy for those who are able to make their own decisions and allow care to be provided in the best interests of those who lack this capacity. The Act rests on five key principles, including*, “a person must be assumed to have capacity unless it is established that they lack capacity”* and *“a person is not to be treated as unable to make a decision merely because he makes an unwise decision”*. The Act requires that an individual is able to understand and retain the information necessary to make a decision, as well as use and weigh that information to arrive at a decision and then be able to communicate the decision once made.

Whilst, according to the Mental Capacity Act, the legal definition of (a lack of) capacity is precise, the application of the definition in clinical practice is less clear cut. Difficulties exist in assessing and operationalising how a patient uses and weighs information and how affective states impact on capacity [10-12]. These areas may be open to influence by individual factors in both the patient and the assessing clinician(s) [13] and it has been argued that capacity determination is intrinsically value laden [14], in line with many aspects of decision making in psychiatry [15]. The potential for a broad range of opinion in individual cases was exemplified by the debate surrounding the decisions made in the case of Kerrie Wooltorton, a 26 year old woman who died in 2007 after drinking antifreeze and refusing life saving treatment having been determined to have capacity by the treating team [16].

Whilst the dimensions of capacity exist on a continuum, determination of mental competence is binary. Thresholds for competence are influenced by how much risk is incurred by the decision being made, with high risk decisions requiring ‘greater’ capacity or margin for error [17] as established in English Law by Lord Donaldson in the case of re T (Adult: Refusal of treatment) [18]. This view is controversial however as it has been suggested that it opens the door for medical paternalism [19]. This debate has a potential impact on practice in assisted suicide - in a survey of US forensic psychiatrists, those with ethical objections to assisted suicide recommended higher thresholds for competence and a more extensive review of the decision [20].

The safeguards proposed in the 2013 Assisted Dying Bill have been informed by the findings published in the Commission on Assisted Dying report, which in turn was informed by the expert evidence given to the Commission. With these ethical, legal and clinical challenges in mind, and considering what might best inform the development of codes of practice on capacity assessment, this study aims to explore how the experts presenting this evidence conceptualised mental capacity for patients requesting assisted suicide and examine these concepts particularly in relation to the principles of the Mental Capacity Act 2005.

## Methods

The Commission on Assisted Dying invited experts drawn from ‘*a wide range of backgrounds*’ [21] to present oral evidence. Thirty seven interviews with 50 experts were video recorded and transcribed. In addition, 13 pieces of written evidence were submitted and published alongside the oral evidence. Two of the authors of this study (AP and MH) submitted one piece of written evidence [22] and gave oral evidence to the Commission [23,24]; these submissions advised caution around the use of mental capacity as a safeguard for assisted suicide. These two pieces of evidence were excluded from analysis; therefore our sample comprised 36 transcripts of oral evidence and twelve pieces of written evidence.

Secondary analysis of the transcripts used a framework approach [25] and comprised four phases: 1) Two researchers (AP and RM) independently familiarised themselves with the data by reading the transcripts and written evidence and watching the videotaped evidence submissions 2) A thematic framework was developed by identifying key issues and concepts present in the data 3) Concepts and themes occurring in the data were discussed and important areas were agreed upon and further discussed with the other members of the research team (MH and TW) before a final agreement was reached 4) Focussed coding of the data was conducted and the main concepts and recurring themes present in the data further defined and refined.

Ethical approval for the study was not required as the data is published in the public domain.

## Results

The 36 oral and 12 written submissions analysed included evidence given by a wide range of experts including 12 medical and social care professionals, eight legal professionals, two persons with disability, four current and former carers/family members, seven academics, three faith group leaders, nine representatives of advocate groups and seven representatives of professional bodies. Of the 15 organisations represented at the Commission, four stated a position in favour of a change in the law to allow assisted suicide, two stated a position against, one stated a position of neutrality and eight did not state a position. Of the clinicians giving evidence, three had specific mental health clinical training (one consultant psychiatrist and two clinical psychologists).

Of the 36 oral evidence submissions, 33 included some reference to mental capacity or issues related to assessment of mental capacity. Of the three submissions that did not refer to mental capacity for patients requesting assisted suicide or related issues, one was from a medical regulatory body which focussed on the current status of assistance of suicide as unlawful and did not enter into discussion regarding safeguarding including mental capacity [submission 20], one was from a medical defence organisation where the discussion focused on doctors’ concerns around the 2010 Director of Public Prosecutions guidance on assisted suicide [submission 14]*,* and one focused on current government policy on end of life care [submission 18]. Table 1 provides a summary of the experts who provided evidence to the Commission.

**Table 1:** Summary of experts presenting evidence to the Commission on Assisted Dying

	**Name**	**Role**	**Organisation represented (if applicable)**	**Type of evidence**	**Further relevant information**
1	DI Adrian Todd	Police Officers	West Mercia Police force	Oral	Investigated the assisted suicide of Daniel James
	DC Michelle Cook				
2	Christine Kalus	Clinical Psychologists in specialist palliative care, Portsmouth City PCT and the Rowans Hospice	British Psychological Society	Oral and written	
	Dr Rebecca Coles-Gale				
3	Joyce Robbins	Patient advocate	Patient concern	Oral	Position in favour of a change in the law to allow assisted suicide
4	Baron Joel Joffe	Lawyer		Oral and written	Proponent of 2004 and 2005 Assisted Dying for the Terminally Ill Bills
		Labour Peer House of Lords			
5	Chris Broad			Oral	Wife Michelle Broad ended her own life in 2010 after being diagnosed with terminal cancer
6	Dr Richard Huxtable	Senior Lecturer and Deputy Director for the Centre of Ethics in Medicine, University of Bristol	Centre for Ethics in Medicine	Oral	Position in favour of no change in the current law
	Dr Martin Curtice	Consultant in old age psychiatry, Holyhill Unit, Birmingham			
7	Martin Green	Chief Executive	The English Community Care Association	Oral	No stated organisational position on assisted suicide
8	Dr Ann McPherson	Chair	Healthcare Professionals for Assisted Dying	Oral and written	HPAD was founded in 2010 and has approximately 400 members
	Dr Ray Tallis	Deputy Chair			
	Professor Joe Collier				
9	Gary Fitzgerald		Action on elder abuse	Oral	No stated organisational position on assisted suicide
10	David Congdon	Head of Campaigns and Policy	MENCAP	Oral	Organisation opposed to change in the law to allow assisted suicide
11	Andrew Copson	Chief executive	British Humanist Association	Oral and written	Organisation in favour of a change in the law to allow assisted suicide
12	Alan Cutkelvin Rees			Oral	Partner ended his life by assisted suicide at Dignitas in 2007
13	Suzy Croft	Senior Social Worker, St John’s Hospice		Oral and written	
14	Dr Stephanie Bown*	Director of Policy and Communications	Medical Protection Society	Oral	Organisation neutral on a change in the law to allow assisted suicide
	Dr Lillian Field*	Medicolegal advisor			
15	Bridget Robb	Development Manager	British Association of Social Workers	Oral and written	No stated organisational position on assisted suicide
16	Pauline Smith	End of life care and dementia lead	NHS West Midlands	Oral	No stated organisational position on assisted suicide
17	Professor Tim Maughan	Consultant Oncologist and Professor of Cancer Studies Cardiff University		Oral	
18	Professor Sir Mike Richards*	National Clinical Director for cancer and End of Life Care	Department of Health	Oral	No stated organisational position on assisted suicide
19	Professor Michael Bennett	Professor of Palliative Medicine, International Observatory on End of Life Care, Lancaster University		Oral	
20	Paul Philip*	Deputy Chief Executive		Oral	
	Jane O’Brien*	Head of Standards and Ethics	General Medical Council		No stated organisational position on assisted suicide
21	Baroness Onora O’Neill	Professor of Philosophy University of Cambridge		Oral	
		Cross bench member House of Lords			
22	Peter Bailey	Trustee Leonard Cheshire disability		Oral	Not representing the organisation-giving evidence in a private capacity
23	Baroness Mary Warnock	Independent cross bench member of the House of Lords.		Oral	Active supporter of legalisation of assisted suicide for the terminally ill.
24	Sarah Wootton	Chief Executive	Dignity in Dying	Oral	Organisation in favour of a change in the law to allow assisted suicide
	Davina Hehir	Head of Policy			
25	Rabbi Danny Rich	Chief Executive Liberal Judaism		Oral	Not representing the organisation-giving evidence in a private capacity
26	Reverend Professor Robin Gill	Professor of Applied Theology, University of Kent		Oral	Not representing the organisation-giving evidence in a private capacity
27	Keir Starmer QC	Director of Public Prosecutions	Crown Prosecution Service	Oral	
28	Professor Clive Seale	Professor of Medical Sociology, Barts and the London School of Medicine and Dentistry		Oral and written	
29	Dr Adrian Tookman	Consultant in palliative medicine, Royal Free Hospital, Hampstead and Medical Director, Marie Curie Hospice.		Oral	
30	Richard Hawkes	Chief Executive	SCOPE	Oral	Organisation opposed to change in the law to allow assisted suicide
	Alice Maynard	Chair			
31	Jane Nicklinson	Wife of Tony Nicklinson, who at the time of the commission was seeking assisted suicide		Oral and written (one written ‘Scheme for assisted death’ and two written statements read out in oral evidence)	
	Saimo Chahal	Solicitor (Representing Tony Nicklinson) with written statements from Tony Nicklinson)			
32	Debbie Purdy	Campaigners for legalisation of assisted suicide		Oral	
	Omar Puente	Husband of Debbie Purdy			
33	Professor Penney Lewis	Professor of Law, School of Law, King’s College London		Oral and written (written evidence co-authored by Genevra Richardson and Roger Brownsword, School of Law, KCL)	
34	Simon Gillespie	Chief Executive	Multiple Sclerosis Society	Oral	No stated organisational position on assisted suicide
35	Lucy Scott Moncrieff	Solicitor, Mental Health Tribunal Judge	Scott-Moncrieff & Associates LLP	Oral and written	
	Robert Robinson	Solicitor			
36	Dr Andrew McCulloch	Chief Executive	Mental Health Foundation	Oral	No stated organisational position on assisted suicide

*No discussion of mental capacity in written or oral evidence.

### Summary of findings

The key themes presented are: 1) The importance of mental capacity in assisted dying legislation; 2) Defining mental capacity, including the boundaries of the concept of mental capacity, 3) The impact of depression on mental capacity 4) Rationality and altruistic assisted suicide and 5) Presumption of capacity. Other themes identified include processes of mental capacity assessment and risks of mental capacity assessment for people requesting assisted suicide and will be presented elsewhere.

### The importance of mental capacity in assisted dying legislation

Where discussed it was unanimously felt that mental capacity should be an important safeguard in any assisted suicide legislation. Assisted suicide for those lacking mental capacity to make the decision was not felt to be appropriate by any of the experts nor was a system allowing advanced decision making to choose assisted suicide for patients who subsequently lost capacity e.g., *‘That decision [in the best interests of someone who lack capacity] of course is not even an issue in the debate about legislation on assisted dying because this legislation is not intended to be of any relevance to people who do not have the capacity to choose and decide’* [Submission 21].

The central importance of mental capacity as a safeguard in assisted suicide legislation was emphasised particularly by those who stated a clear position in support of assisted suicide, for example, representatives of Health Professionals for Assisted Dying stated the importance of mental capacity five times during their oral submission and representatives of Dignity in Dying stated this four times e.g., *‘I hope we have made it clear that we would want any assisted dying process to include a rigorous process to ensure that somebody only could do this if they meet the criteria, including capacity’* [Submission 24].

### Definition and boundaries of the concept of mental capacity

Whilst the majority of experts made reference to mental capacity in their submissions, few explicitly defined it. Only in seven submissions were any direct questions asked by the panel regarding capacity, and none of the experts were asked to give a definition. For those who did define mental capacity, it was described variously as being ‘*of sound mind’* , possessing *‘rational autonomy’* having a ‘*robust wish’* , ‘*full understanding’* and being *‘able to make their own mind up…’.* The terms ‘*capacity’* , ‘*competence’* and in one case ‘*consent’* appeared to be used with the same intended meaning. There did not appear to be a clear distinction between the definitions used by clinicians compared with non-clinicians, between professional groups, or between those in favour of or opposed to assisted suicide.

The findings showed that where described, conceptualisations of mental capacity were on a spectrum. At one end was a tightly defined ‘cognitive’ or’ intellectual’ conceptualisation, ,whilst at the other was a broader conceptualisation involving a number of components, e.g., *‘…it does show a fundamental flaw with our thinking about capacity - that it’s clearly a sort of intellectual function, and it is not. It’s a holistic function, a combination of the intellectual, emotional, perceptual and so on and how that reasoning comes together’* [Submission 36].

Of the 10 submissions in which an explicit conceptualisation of mental capacity was described, five presented a cognitive/intellectual conceptualisation and five a broader conceptualisation. Of the five presenting a cognitive/intellectual conceptualisation, three stated a position strongly in favour of the legalisation of assisted suicide *[Submissions 3, 4, 8]* and of those describing a broader conceptualisation, none stated a personal or organisational position regarding the legalisation of assisted suicide *[Submissions 2, 9, 21, 26, 36].*

### The impact of depression on mental capacity

The range of boundary conceptualisations was particularly illustrated when examining the interface between mental state and mental capacity, with the relationship between the decision to request assisted suicide and depression a particular area of inconsistency. There appeared to be a prevailing sense that this was a difficult area in mental capacity determination and in particular that separating the normal emotional response to life limiting illness and an abnormal mental state is problematic. There was inconsistency in the views presented as to whether depressive symptoms (either as a clinical depressive syndrome or reactive depressive symptoms in response to terminal illness) would impact negatively on one’s capacity to make this decision e.g., *‘Now major depression in itself, if you apply the Mental Capacity Act, does not automatically mean you lack capacity, but it’s highly likely to influence your decision-making’* [Submission 6] vs *‘Obviously you expect that a person who is dying might feel quite sad about that and that’s a different thing to depression, then that’s a different thing again to whether or not somebody has capacity’* [Submission 25].

It was unclear from the evidence what severity of depression would be considered likely to have an impact upon capacitous decision making and how this might be determined. Some felt that any depression might impact upon decision making capacity, *‘Yes it can do, I mean depression can affect one’s capacity to make decisions or to behave emotionally, cognitively, behaviourally, in all sorts of ways’* [Submission 2], whilst others thought that a finding of depression would almost always be consistent with capacity if only a cognitive test of capacity were applied, e.g., *‘…a majority of depressed patients will meet that test [the Mental Capacity Act test of capacity], so we can’t rely on those requests related factors to deal with the victim who is suffering from depression or some other mental disorder’* [Submission 33].

### Rationality and altruistic assisted suicide

The findings also showed that for a number of experts, wider concepts including autonomy, rationality, voluntariness and motivating factors behind the decision were felt to be integrally related to mental capacity and would need to be explored as part of a comprehensive assessment of decision making ability. This was illustrated particularly in the range of opinions on the concept of ‘altruistic assisted suicide’. Some experts felt that it was appropriate and consistent with capacity to decide that one’s continued life is a burden to others and decide to seek assisted suicide with the intention of relieving them of that burden e.g., *‘I think there are all sorts of pressures that are going to influence your decision and some of them get sort of hauled out as how this is terrible, you feel you’re a burden. Well, I don’t want to be a burden; I think an altruistic choice is a perfectly reasonable choice’* [Submission 3]*,* whilst for others, rationality appeared to be synonymous with capacity and was felt to be incompatible with choosing assisted suicide to reduce burden on others, *‘When is the person making the decision rationally, or because they feel they don’t want to be a burden on their family?’* [Submission 24] Other experts felt that altruistic motives were not acceptable as motivating factors and if identified should be a barrier to assisted suicide e.g., *‘…in a sense, what you’re looking for here is that the person’s reasons are selfish, that they’re doing it for themselves, not for somebody else’* [Submission 35].

### Presumption of capacity

Whilst presumption of capacity was cited by a number of experts as an important cornerstone in thinking about mental capacity assessment e.g., *‘… obviously the starting point would be of presumed capacity under the Mental Capacity Act’* [Submission 6]*,* a number of experts felt that a formal assessment of capacity should take place for every patient making a request e.g., ‘*I hope we have made it clear that we would want any assisted dying process to include a rigorous process to ensure that somebody only could do this if they meet the criteria, including capacity*’ [Submission 24].

There appeared to be a lack of consistency in whether the assessment was required to prove that the individual possessed or lacked capacity for the decision: possession of mental capacity was cited as an inclusion criterion for assisted suicide by some, e.g., *‘I mean it is quite clear to us that there are some fundamental points. Firstly, making sure that the person truly does have capacity’.* [Submission 24] whilst lack of capacity was cited as an exclusion criterion by others: *‘So this would exclude, for example…assisted dying for someone who doesn’t have mental capacity…* ‘[Submission 8].

There was variability in the perceived relationship between presumption of capacity and the appropriate use of Mental Capacity Act as an assessment framework as shown in Table 2. Only two experts who advocated for the use of the Mental Capacity Act made a clear statement that mental capacity for assisted suicide should be presumed; a further nine either explicitly stated that they would not presume capacity, made contradictory statements within their submission or made unclear statements about whether capacity should be presumed. Of the five experts who made clear statements that capacity should be presumed, two advocated the use of the Mental Capacity Act, one made a clear statement that the Mental Capacity Act was inadequate for use in assessing capacity in this circumstance, one was unclear on whether the Mental Capacity Act was a suitable framework and one did not discuss the assessment framework in their submission.

**Table 2:** Relationship between presumption of capacity and endorsement of the use of the Mental Capacity Act 2005

		**Presumption of capacity**				
		**Yes**	**No**	Unclear/both*	**Not discussed**	**Total**
Mental Capacity Act	Yes	2	1	4	2	9
	No	1	0	0	0	1
	Unclear	1	0	2	0	3
	Not discussed	1	1	1	17	20
	Total	5	2	7	19	33

*Within one expert submission there are statements which suggest both presumption and non-presumption of capacity.

## Discussion

The report published by the Commission on Assisted Dying advocates a change in the law to allow assisted suicide and cites possession of the mental capacity to make a request as a ‘*key element that should underpin a safeguarded framework for assisted dying’*; but this assertion is based on evidence that presents unclear and inconsistent concepts of mental capacity and little discussion about the standards and frameworks that should be used to assess this capacity.

Within the submitted evidence there were two key areas of consistency among the experts. Firstly that mental capacity should be a central safeguard and secondly that advance decision making for those likely to lose capacity in the future is not appropriate for assisted suicide: capacity should be present at the time the decision is being made. But between and sometimes within expert submissions, there was a lack of consistency in the definition and boundaries of the concept of mental capacity, and the interface of capacity with other areas that might have a bearing upon its determination, particularly motivation, voluntariness, autonomy, rationality and the presence and severity of mental disorder, specifically depression.

The Commission on Assisted Dying has strongly recommended that any assisted suicide legislation be closely regulated and safeguarded. Mental capacity determination as set out in the Mental Capacity Act 2005 has surface validity for fulfilling this safeguarding role but deeper exploration of the evidence informing these recommendations shows that the ways in which mental capacity is conceptualised are diverse. Several of the experts expressed ideas that were not consistent with the principles of the Mental Capacity Act, particularly the presumption of capacity.

The Act makes it clear that if there is no demonstrable disorder of mind or brain then the patient is free to make whatever decision they choose regardless of whether this is wise, unwise or no decision is made at all. In order for the Commission to recommend assessment of capacity for all patients there may be an implicit normative judgment about the decision to request assisted suicide being strongly indicative of a disorder of mind or brain, but this potentially introduces a problem-that the decision itself implies that capacity may be impaired but in order to proceed with assisted suicide it must be demonstrated that it is not. The conceptual shift toward demonstrating presence rather than lack of capacity, reflected in the recommendation from the Commission that capacity be assessed formally in all cases is also potentially problematic because within the Mental Capacity Act there is no clear definition of mental capacity (only a lack of capacity) and no clarity or guidelines on what would constitute sufficient mental capacity to decide to undergo assisted suicide.

The Mental Capacity Act 2005 test of capacity only applies to England and Wales. Internationally, most jurisdictions base their capacity laws on a ‘functional’ approach which is decision and time specific rather than ‘outcome’ or ‘status’ based approach and capacity is presumed; [26] however, different jurisdictions use different components for capacity determination; for example in the US, the capacity test is based on national case law and evaluates dimensions of ‘understanding’ , ‘appreciation’ , ‘reasoning’ and ‘expressing’ a choice. Also, there are a number of instruments used to assess capacity [27] for example the Macarthur Competency Assessment Tool (MacCAT-T) developed in the US [28].

Of the jurisdictions internationally where assisted suicide is legal, all include mental capacity as part of their safeguards [29] but only the Oregon and Washington statutes give an explicit definition of mental capacity [30]. Guidelines for mental health professionals accompanying the Oregon Death with Dignity Act (DWDA) outline the capacity evaluation process, but these acknowledge that this process is difficult, especially in determining the impact of mental disorders on decision making ability [31].

Challenges in mental capacity determination are not unique to the situation of assisted suicide. In healthcare, both refusals (e.g., refusal of further life sustaining treatment) and requests (e.g., requests for gender reassignment or living organ donation) require a determination of mental capacity which can often involve detailed and wide-ranging assessment in order to reach a satisfactory conclusion. Assessment of factors such as motivation and voluntariness will often form part of a comprehensive assessment of decision making in these circumstances.

In her paper examining mental capacity using an anthropological approach, Doorn [32] argues that the available literature focuses on criteria for the assessment of competence without elaborating on what it is to be competent or incompetent to make a decision. She describes ‘thin’ and ‘thick’ conceptualisations of capacity which correspond to a more cognitive conceptualisation based on ‘negative’ autonomy (self determination with freedom from the interference from others) and a richer conceptualisation which acknowledges values (both of the patient and clinician) and is based on ‘positive’ autonomy (the potential for self development and fulfilment). She argues that assessment tools used to measure capacity have their roots in a ‘thin’ conceptualisation which does not acknowledge the ‘value ladenness’ of capacity decisions but rely on narrower cognitive abilities. This view is not without criticism [33,34] but is echoed by other authors who argue that a value neutral or value free conceptualisation of capacity is potentially problematic in practice [14] and that capacity assessment is inherently normative and irreducible to a set of objective criteria [35]. The findings of this study showed that a cognitive conceptualisation was more frequently endorsed by those strongly in favour of assisted suicide which would appear to be consistent with the value of self-determination, but among the experts there were a number of normative judgements being made about reasons for requesting assisted suicide e.g. being a burden on others. Ideas about ‘reasonable’ and ‘unreasonable’ reasons for requesting assisted suicide further emphasise the subjectivity potentially inherent in the process.

The interface between mental state and mental capacity continues to present challenges and this issue is far from resolved. Even within an assessment framework emphasising cognitive elements of mental capacity, depression may have a significant bearing in terms of their ability to use and weigh the relevant information, but how far this might be tolerated and the patient still be found competent to make the decision to request assisted suicide remains unclear.

Depression is common in palliative care [36] and desire for hastened death is strongly associated with depression in palliative populations [37]. In Oregon it has been shown that depression is not always appropriately identified in patients requesting assisted suicide [38]. There is evidence to suggest that treatment of depression can reduce the wish for hastened death [39] and that antidepressants are effective in patients with life threatening illness [40].

### Strengths and limitations

This study analysed data that were not originally gathered for the purpose of examining concepts of mental capacity. This could be seen as both a limitation and a strength. Because the study used secondary analysis of these data there was no opportunity to further examine concepts or directly compare similar data. Had they been interviewed with mental capacity as the main focus, the experts may have presented different ideas and perspectives and different conclusions may have been reached. However, the strength of these data is that they provided an opportunity to examine the experts’ ‘naturalistic’ ideas about capacity and to analyse the points of convergence with and divergence from current legal, clinical and philosophical constructs.

The experts presenting to the Commission were invited by the Commissioners because of particular interest or expertise in areas related to the subject being examined. Few were experts in mental health and even fewer experts in mental capacity determination. This sample can therefore not be considered to be representative of current thinking about mental capacity but the responses do show a range of ideas about mental capacity from several different backgrounds, disciplines and ethical standpoints.

The authors acknowledge that one’s ethical standpoint on the legalisation of assisted suicide can have a bearing on individual ideas about mental capacity, particularly the standard required for possession of capacity [20]. Reflexivity is an important element of analytic rigour in qualitative methodology [41] which allows the research to be placed in appropriate context so that conclusions can be judged in light of this context. The researchers analysing and reporting this data have a particular interest in mental capacity assessment and three of the authors (AP, MH and RMC) are clinicians who frequently assess mental capacity as part of their roles and are familiar with the challenges of applying the legal framework of the Mental Capacity Act 2005 in complex clinical situations including end of life decision making. We take the position that legalisation of assisted suicide is a matter for society to decide through due parliamentary process but AP and MH have previously expressed concerns about mental capacity as a safeguard in assisted dying legislation in part due to a concern about the potential for subjectivity and normativity in the process and outcome of clinical assessment [10]. One of the authors (MH) has undertaken a review of reliability in mental capacity assessment and found that this is good when rigorous assessment procedures are applied but less so for less structured clinical assessments [42]. MH has also commented previously on the difficulties of clearly defining mental capacity due to its varying conceptualisation as a legal, clinical or social construct and differing definitions across jurisdictions [17].

## Conclusions

The Mental Capacity Act was originally conceived as a statutory framework to protect those who lack capacity to make decisions for themselves and provide a mechanism by which others can make decisions on their behalf in their best interests. Clinically the model fits quite well in situations where patients are refusing proposed interventions as the Act makes statements about an individual ‘lacking’ rather than having capacity for the decision that is being made, therefore mapping more closely onto Banner’s ‘thin’ rather than ‘thick’ conceptualisation of mental capacity. Capacity decisions for ‘requests’ including for assisted suicide appear to have their origins more in a ‘thick’ conceptualisation of capacity which as Banner suggests may not be fully served by the current legal structures. In addition the Act stipulates some explicit exclusions to its use (in part 3, section 62), one of which is in the operation of the Suicide Act 1961 [43] (assisting suicide); therefore the proposition of using the Mental Capacity Act for capacity assessment for patients requesting assisted suicide potentially presents a legal as well as a clinical problem.

The tension between differing conceptualisations of mental capacity presents difficulties for policy makers, lawyers and clinicians. If, as the Commission recommends, we consider a model of capacity assessment that seems implicitly to presume non capacity and place the burden of proof on determining that capacity is present rather than absent, then it is difficult to see how the procedures set out in the Mental Capacity Act 2005 can be applicable. The question is raised as to whether it is Mental Capacity Act ‘mental capacity’ or rather a broader set of faculties that the Commission (and subsequent Bill) envisages, which have an inherent value ladenness that may render the process more subjective, and arguably provide a less reliable safeguard in the process.

The experts presenting evidence to the Commission on Assisted Dying were inconsistent in their conceptualisations of mental capacity as it relates to assisted suicide. Before mental capacity can be placed so centrally as a safeguard in the process, discussion needs to take place about what exactly is meant by the term ‘mental capacity’ in the new Assisted Dying Bill. Only then can decisions be made as to whether it meets the need for which it has been identified and whether current legal frameworks are able to support clinicians in determining capacity for this group.

## Competing interests

All authors declare: no support from any organisation for the submitted work; no financial relationships with any organisations that might have an interest in the submitted work in the previous three years; MH and AP presented evidence to the Commission on Assisted Dying and advised caution in considering mental capacity as a safeguard in assisted dying legislation, MH was on the Royal College of Psychiatrists working group on Assisted Dying, and during a consultation run by the College, voiced concern about a change in the law based on his experience of caring for people requesting assisted dying.

## Authors’ contributions

AP, TW and MH devised and planned the study. AP and RM planned and performed data analysis supervised by TW and MH. AP drafted the manuscript and all authors contributed to the submitted version. AP is guarantor. All authors read and approved the final manuscript.

## Pre-publication history

The pre-publication history for this paper can be accessed here:

http://www.biomedcentral.com/1472-6939/15/32/prepub
